# Supplementary material for: Important Aspects Influencing Delivery of Serious News in Pediatric Oncology: A Scoping Review
Source: Children (Basel). 2021 Feb 22;8(2):166. doi: 10.3390/children8020166 (PMC7926514; doi:10.3390/children8020166)
Supplement: Supplementary file 1 [file children-08-00166-s001.pdf]

Table S1. Search Formula.

Database		Search Formula
EMBASE (OvidSP)		(Child and (Oncology or Neoplasm) and ("Interpersonal Communication" or "Medical Information" or "Doctor Patient Relation")).sh. and (Czech or English or French or Slovak).lg. and limit to yr="1990 -Current" and limit to type="Journal: Article"
Scopus		(KEY ((child AND ("Medical Oncology" OR neoplasm*) AND (communication OR "Health Communication" OR "Truth Disclosure" OR "Physician-Patient Relations"))) AND LANGUAGE ((czech OR english OR french OR slovak))) AND DOCTYPE (ar) AND PUBYEAR > 1989
Cochrane Library (OvidSP – EBM)	1	(Child and ("Medical Oncology" or Neoplasms) and (Communication or "Health Communication" or "Truth Disclosure" or "Physician-Patient Relations")).sh. and (Czech or English or French or Slovak).lg. and limit yr="1990-2017" and limit to type="Journal Article")
	2	(Child and (Oncology or Neoplasm*) and (Communication or "Health Communication" or "Truth Disclosure" or "Physician-Patient Relations")).ab. and (Czech or English or French or Slovak).lg. and limit to yr="1990-Current" and limit to type="Journal Article"
Cochrane Library<br><br>via search engine on http://www.cochranelibrary.com/	1	'(Child and ("Medical Oncology" or Neoplasms) and (Communication or "Health Communication" or "Truth Disclosure" or "Physician-Patient Relations")) in Title, Abstract, Keywords in Cochrane Reviews'<br><br>→using MeSH

	2	'(Child and (Oncology or Neoplasm) and ("Interpersonal Communication" or "Medical Information" or "Doctor Patient Relation")) in Title, Abstract, Keywords in Trials'<br>→using Embase subject headings .
Medvik (Bibliographia Medica Checoslovaca)		MeSH: dítě AND MeSH: ("lékařská onkologie" or nádory) AND MeSH: (komunikace or "zdravotnická komunikace" or "sdělení pravdy" or "vztahy mezi lékařem a pacientem")<br>Limitery:vydáno: 1990-2017; typ dokumentu: články
PsycINFO		MA Child AND MA ((Communication OR "Physician-Patient Relations" OR "Truth Disclosure" OR "Health Communication")) AND MA (("Medical Oncology" OR Neoplasms)) AND LA ((Czech OR Slovak OR English OR French))

Table S2: PRISMA ScR Checklist

**Section**	**Item**	**PRISMA-ScR Checklist Item**	**Page**
**Title**	1	Identify the report as a scoping review.	Title page
**Abstract**<br>Structured summary	2	Provide a structured summary that includes (as applicable) background, objectives, eligibility criteria, sources of evidence, charting methods, results, and conclusions that relate to the review questions and objectives.	Abstract
**Introduction**<br>Rationale	3	Describe the rationale for the review in the context of what is already known. Explain why the review questions or<br>objectives lend themselves to a scoping review approach.	2-3
Objectives	4	Provide an explicit statement of the questions and objectives being addressed with reference to their key elements (for example, population or participants, concepts, and context) or other relevant key elements used to conceptualize the review questions or objectives.	4
**Methods**<br>Protocol and registration	5	Indicate whether a review protocol exists; state if and where it can be accessed (for example, a Web address); and if available, provide registration information, including the registration number.	4
Eligibility Criteria	6	Specify characteristics of the sources of evidence used as eligibility criteria (for example, years considered, language, and publication status), and provide a rationale.	4-5
Information Sources	7	Describe all information sources in the search (for example, databases with dates of coverage and contact	5

		with authors to identify additional sources), as well as the date the most recent search was executed.	
Search	8	Present the full electronic search strategy for at least 1 database, including any limits used, such that it could be repeated.	5-6 Suppl. Table S1
Selection of sources of evidence	9	State the process for selecting sources of evidence (that is, screening and eligibility) included in the scoping review.	6
Data charting process	10	Describe the methods of charting data from the included sources of evidence (for example, calibrated forms or forms that have been tested by the team before their use, and whether data charting was done independently or in duplicate) and any processes for obtaining and confirming data from investigators.	6-7
Data Items	11	List and define all variables for which data were sought and any assumptions and simplifications made.	6-7 Suppl. Table S2
Critical appraisal of Individual sources of evidence	12	If done, provide a rationale for conducting a critical appraisal of included sources of evidence; describe the methods used and how this information was used in any data synthesis (if appropriate).	n/a
Summary measures	13	This item from the original PRISMA is not applicable for scoping reviews because a meta-analysis is not done (that is, summary measures are not relevant).	n/a
Synthesis of results	14	Describe the methods of handling and summarizing the data that were charted.	8-9
Risk of bias	15	This item from the original PRISMA is not applicable for scoping reviews because the scoping review method is not intended to be used to critically appraise (or appraise the risk of bias of) a cumulative body of evidence.	n/a
Additional analyses	16	This item from the original PRISMA is not applicable for scoping reviews because additional analyses, including sensitivity or subgroup analyses and meta-regression, are not done.	n/a
**Results** Selection of sources of evidence	17	Give numbers of sources of evidence screened, assessed for eligibility, and included in the review, with reasons for exclusions at each stage, ideally using a flow diagram.	7 Figure 1
Characteristics of sources of evidence	18	For each source of evidence, present characteristics for which data were charted and provide the citations.	8 Table 1
Critical appraisal within sources of evidence	19	If done, present data on critical appraisal of included sources of evidence (see item 12).	8
Results of individual sources of evidence	20	For each included source of evidence, present the relevant data that were charted that relate to the review questions and objectives.	Table 1
Synthesis of results	21	Summarize or present the charting results as they relate to the review questions and objectives.	8-9
Risk of bias across studies	22	This item is not applicable for scoping reviews. See explanation for item 15.	n/a
Additional analyses	23	This item is not applicable for scoping reviews. See explanation for item 16.	n/a

**Discussion**<br>Summary of evidence	24	Summarize the main results (including an overview of concepts, themes, and types of evidence available), link to the review questions and objectives, and consider the relevance to key groups.	10-13
Limitations	25	Discuss the limitations of the scoping review process.	13-14
Conclusions	26	Provide a general interpretation of the results with respect to the review questions and objectives, as well as potential<br>implications or next steps.	14-15
**Funding**	27	Describe sources of funding for the included sources of evidence, as well as sources of funding for the scoping review.<br>Describe the role of the funders of the scoping review.	15

Table S3: Codes Identified in Each Participant´s Category

PHYSICIAN (N=27)	PARENT (N=45)	SIBLING (N=5)	PATIENT (N=17)
close contact	empathy	presence of sibling	empathy
parents´ trust	honesty	respect	honesty
colleague support	being open	amount of info	support
loneliness	acknowledge uncertainty	timing	encouragement
debriefing	respect	sibling´s age	respect
timing - no delay	assurance		adolescents
culture aspects	straight		clarity of info
lack of time	"never-give-up" attitude		amount of info
training	appropriate manner setting		vocabulary
preparation in advance	warning first		repeating info
vulnerability	chance to preparation		timing
empathy	language		patient´s presence
honesty	proper vocabulary		access to answers
avoid identification	parents ´ control		parental advocacy
sense of responsibility	amount of info		honesty
protecting the child	length of interview		rely on parent
emotional state	physician´s expertise		continuity of care
conflict of interests	clarity		
parental burden concerns	avoid contradictory info		
maintaining hope	written information		
respect towards parents	repeated info		
aggressiveness	patient´s presence/involvement		

expertise	repeating info		
guidelines	physician´s availability		
competency	specific info		
diagnosis	privacy		
severity	timing		
	end of curative TX		
	no delay in informing		
	child - protecting		
	child expertise		
	lack of professional expertise		
	trustful relationship		
	duty		
	no hurry		
	others´ support / involvement		
	presence of sibling		
	presence of other staff		
	presence of other family members		
	close contact with physician		
	severity		
	impending death		
	curative TX		
	continuity of care		
	child´ s personality		
